# Time- and depth-wise trophic niche shifts in Antarctic benthos

**DOI:** 10.1371/journal.pone.0194796

**Published:** 2018-03-23

**Authors:** Edoardo Calizza, Giulio Careddu, Simona Sporta Caputi, Loreto Rossi, Maria Letizia Costantini

**Affiliations:** 1 Department of Environmental Biology, Sapienza University of Rome, Rome, Italy; 2 CoNISMa-Consorzio Nazionale Interuniversitario per le Scienze del Mare, Rome, Italy; University of Waikato, NEW ZEALAND

## Abstract

Climate change is expected to affect resource-consumer interactions underlying stability in polar food webs. Polar benthic organisms have adapted to the marked seasonality characterising their habitats by concentrating foraging and reproductive activity in summer months, when inputs from sympagic and pelagic producers increase. While this enables the persistence of biodiverse food webs, the mechanisms underlying changes in resource use and nutrient transfer are poorly understood. Thus, our understanding of how temporal and spatial variations in the supply of resources may affect food web structure and functioning is limited. By means of C and N isotopic analyses of two key Antarctic benthic consumers (*Adamussium colbecki*, Bivalvia, and *Sterechinus neumayeri*, Echinoidea) and Bayesian mixing models, we describe changes in trophic niche and nutrient transfer across trophic levels associated with the long- and short-term diet and body size of specimens sampled in midsummer in both shallow and deep waters. Samplings occurred soon after the sea-ice broke up at Tethys Bay, an area characterised by extreme seasonality in sea-ice coverage and productivity in the Ross Sea. In the long term, the trophic niche was broader and variation between specimens was greater, with intermediate-size specimens generally consuming a higher number of resources than small and large specimens. The coupling of energy channels in the food web was consequently more direct than in the short term. Sediment and benthic algae were more frequently consumed in the long term, before the sea-ice broke up, while consumers specialised on sympagic algae and plankton in the short term. Regardless of the time scale, sympagic algae were more frequently consumed in shallow waters, while plankton was more frequently consumed in deep waters. Our results suggest a strong temporal relationship between resource availability and the trophic niche of benthic consumers in Antarctica. Potential climate-driven changes in the timing and quality of nutrient inputs may have profound implications for the structure of polar food webs and the persistence of their constituent species, which have adapted their trophic niches to a highly predictable schedule of resource inputs.

## Introduction

Climate change is expected to affect the resource-consumer interactions providing stability in aquatic food webs [1]. The effects will be particularly pronounced in polar marine ecosystems, where modifications in temperature and sea-ice coverage will affect the quantity and timing of resource supply to consumers [2–5]. Diversity and the temporal fluctuation of resource inputs are key ecosystem properties, promoting the stability of food webs [6–8]. This is particularly true when generalist consumers are able to feed across multiple energy channels whose availability can vary in space and time (e.g. the detritus and the herbivore pathways), guaranteeing continuous energy transfer to higher trophic levels [7,9,10]. In polar habitats, inputs of nutrients from sediment and primary producers represent complementary food sources, whose availability dramatically changes with the season [11]. In these habitats, organisms have adapted to such pulsed resource supply by concentrating foraging and reproductive activity in the summer months, when inputs from pelagic and sympagic producers increase [2,12,13]. This enables the persistence of biodiverse food webs despite the extremely limiting environmental conditions. While physiological adaptations to physical constraints have been widely addressed, the trophic-functional mechanisms promoting resource partitioning between organisms and nutrient transfer across trophic levels are less well understood [14]. Thus, our understanding as to whether and how future spatial and temporal changes in the supply of resources will translate into changes in polar food webs is limited [15–17].

In Antarctica, C and N stable isotope analyses (SIA) have been used to characterise marine taxa [18–20]. Isotopic signatures in a consumer’s tissues reflect those in its food sources [21–23], and have been shown to differ between guilds of primary producers, i.e. plankton and benthic and sympagic algae [18]. Thus, SIA makes it possible to infer resource use by consumers [22–25]. In addition, comparison of portions characterised by slow (e.g. muscle) and fast (e.g. gut content) turnover rates can be suitable for describing variations in the diet of organisms in seasonally constrained ecosystems [18,26]. However, the description of trophic links in benthic Antarctic habitats is strongly limited by logistical problems. Only one study has used linear isotopic mixing models to describe the diet of shallow-water benthic consumers [18], and no isotopic descriptions are available for benthic species in medium-depth waters, where the majority of Antarctic biodiversity lies [27]. Similarly, no attempts have been made to translate information on resource use into measures of population niche width and the uniformity of energy flows in the food web. Thus, theories that have been widely used elsewhere to explain patterns of biodiversity organisation along resource gradients (i.e. Niche Theory [28] and Optimal Foraging Theory [23,29]) remain untested for benthic polar habitats, limiting ecological forecasts in these regions.

In this study we addressed the trophic niches of two key Antarctic benthic consumers, *Adamussium colbecki* Smith (Bivalvia) and *Sterechinus neumayeri* Meissner (Echinoidea), and related implications for nutrient transfer within the food web. These species (i) belong to the two most widely represented feeding guilds within Antarctic benthic communities (i.e. facultative filter feeders and omnivorous deposit feeders), (ii) are widely distributed and often found in association along the Antarctic coasts (S1 Fig), and (iii) make an important contribution to secondary productivity in the areas they colonise [27,30–33]. By means of C and N isotopic analyses of organisms sampled in midsummer and Bayesian mixing models, we described differences in the long-term (by analysing soft tissues) and short-term (by analysing gut contents) trophic niches associated with depth and body size at Tethys Bay, an area characterised by extreme seasonality in sea-ice coverage and productivity in the Ross Sea.

Based on the expected increase in the contribution of sympagic algae and plankton to the diet of consumers in summer, we quantified temporal variations in (i) isotopic niche structure, (ii) diet composition and (iii) the degree of coupling of energy channels in the food web arising from consumers feeding on multiple energy pathways (i.e. sediment, benthic algae, plankton and sympagic algae). We compared such variations in both shallow (15–25 m) and deep (50–150 m) waters. In accordance with optimal foraging theory, which expects consumers to specialise when in the presence of high resource availability [29], and field evidence from temperate habitats [23,34], we hypothesised higher trophic specialism in the short term, and thus less marked coupling of distinct energy channels in the food web, following the expected input of additional food [35–37].

## Materials and methods

### Ethics statement

The present study is part of the PNRA 2010/A1.07 project. No protected species were involved. The sampling activity and target species were agreed with the PNRA (Italian National Antarctic Research Program), which issued permits to collect samples in the study area on behalf of the Italian Ministry of Foreign Affairs (Permit: N°1-PNRA 2010/A1.07, issued in compliance with the "Protocol on Environmental Protection to the Antarctic Treaty", Annex II, art.3).

### Sampling area and target species

Samplings were performed at Tethys Bay, in the area of Terra Nova Bay (Ross Sea) (74°41’40”S 164°03’22”E), during the third week of January 2013. The bay measures 3 km from the inner to the outer limit. It is connected with the open waters of the polynya of Terra Nova Bay, which, after the onset of phytoplankton bloom, allows for the advection of pelagic material even during ice-covered periods [18,38,39]. Sea-ice coverage and primary productivity in the bay are characterised by marked seasonality, with periods of complete absence of ice coverage during summer and phytoplankton blooms typically observed in January [15]. Further information on the study area can be found in [18,39,40]. Target species were the bivalve *Adamussium colbecki* and the sea urchin *Sterechinus neumayeri*. These species are abundant in the study area and are able to vary their diet in response to changes in resource availability [18,30–33]. *A*. *colbecki* is a facultative filter feeder, while *S*. *neumayeri* is an omnivorous opportunistic feeder, able to switch from grazing to scavenging.

Sampling was performed along two linear transects in order to collect specimens in the depth ranges of 15–25 m (hereafter “shallow”) and 50–150 m (hereafter “deep”) (S2 Fig). In shallow waters, sampling was performed by hand by scuba divers, while sampling in deep waters was performed by dredging. The shallow and deep sampling transects were 700 m apart, at a similar distance from the edge of the sea ice. Sea-ice broke up in our sampling sites three days before sampling, evident sea-ice melting and cracking in the bay having started a week earlier. The edge of the sea-ice was only 0.3 km from our sampling transects six days before sampling and ~1 km two weeks before, when sea-ice outside the bay broke up completely.

Using a digital calibre, test diameter and height were measured for *S*. *neumayeri*, while shell height, length and width were measured for *A*. *colbecki*. Specimens were dissected and gut contents collected. This made it possible to compare isotopic signatures and diet composition over the short term (gut content) and long term (soft tissue) [18], when dredging in deep waters was not possible due to sea-ice coverage. The number of *A*. *colbecki* individuals considered for isotopic analysis of muscle and gut contents was n = 32 (14 in shallow and 18 in deep waters) in both cases, while for *S*. *neumayeri* it was n = 42 for muscle (25 in shallow and 17 in deep waters) and n = 33 for gut contents (21 in shallow and 12 in deep waters). For isotopic analyses, white muscle tissue from *A*. *colbecki* and the peristomal membrane of *S*. *neumayeri* were collected. Given that they have slower turnover rates than other tissues (e.g. blood, gonads), these tissues are expected to reflect the diet assimilated over the several weeks or even months before sampling [18,21]. This should be particularly true for Antarctic organisms, whose slow metabolism allows us to assume that the isotopic signatures of soft tissues are indicative of food assimilated well before sea-ice broke up in the study area. In order to characterise the short-term diet, gut contents were carefully collected under a stereoscope in order to avoid gut tissue. For *S*. *neumayeri*, in order to consider recently ingested food only, gut contents were collected considering the first tract of the gut (between one third and one half of the total gut length). For *A*. *colbecki*, the digestive gland was separated intact from the rest of the body, partly incised under a stereoscope, and the contents placed in a sterile petri dish. The gland was then completely dissected within a second petri dish and any remaining contents were examined under a stereoscope and collected.

**Fig 1:**
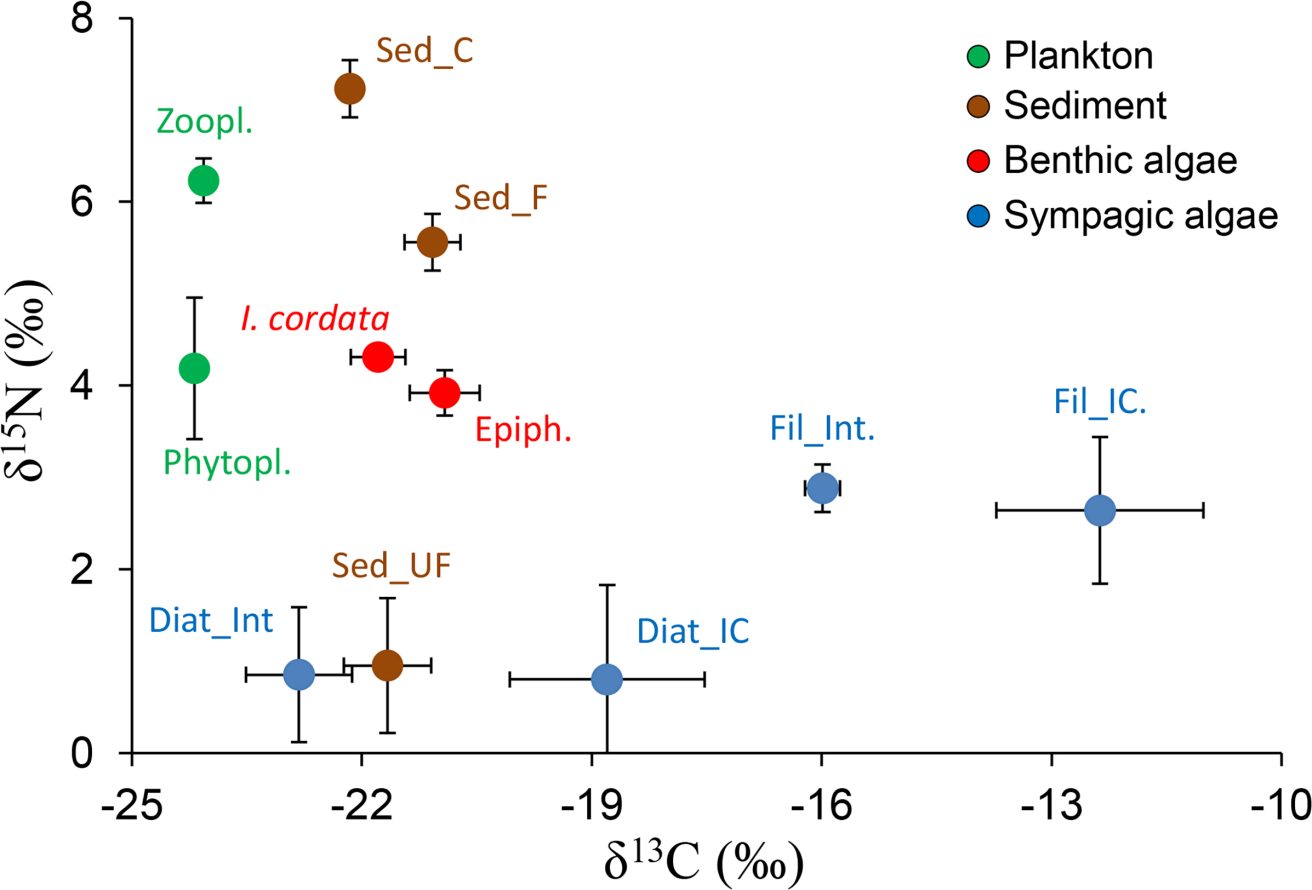
Isotopic signatures of food sources. Mean (±S.E.) δ^13^C and δ^15^N values of basal food sources at Tethys Bay (Ross Sea, Antarctica). Coarse (C), fine (F) and ultra-fine (UF) refer to different size fractions of organic matter in sediment (Sed). Resource guilds are indicated with colours, i.e. blue: sympagic resources; green: pelagic resources; brown: organic matter in sediments; purple: benthic primary producers.

Resources potentially contributing to the diet of *A*. *colbecki* and *S*. *neumayeri* were collected (Fig 1 and S1 Table). These included (i) benthic primary producers (the red algae *Iridaea cordata*, *Phyllophora antarctica* and associated epiphytic diatoms [41]); (ii) organic matter in sediments (coarse: >1 mm; fine: between 1 mm and 0.56 mm; ultra-fine: <0.56 mm); (iii) pelagic production (phytoplankton and zooplankton); (iv) sympagic primary production (microscopic and filamentous algae growing both within the ice-core and at the interface between sea-ice and water).

Sympagic algae were collected in spring (i.e. November), before the sea-ice broke up, while phytoplankton and zooplankton were collected in both spring and summer (i.e. January). Benthic macroalgae and associated epiphytes were collected in both spring and summer in shallow waters, while sampling by dredging in deep waters was not possible in spring. Given that (i) the isotopic signatures of macroalgae and epiphytes in shallow waters were highly similar between seasons, (ii) the signatures of macroalgae strongly resembled those obtained from specimens sampled in shallow waters in Tethys Bay in 2003 [18], and (iii) the composition of epiphytic diatoms associated with *P*. *antarctica* has been shown to be conserved over time and across depths in our study area [41], the isotopic signal of these basal resources was assumed to be highly conserved, and the δ^13^C and δ^15^N values obtained in summer were also used for the determination of the long-term diets of consumers in deep waters. Sediments were collected in both shallow and deep waters in summer. Since spring sediments were not available for isotopic analysis, we considered sediment δ^13^C and δ^15^N measured in summer for the reconstruction of both long- and short-term diet. Indeed, Antarctic sediments are known to act as food deposits with very slow turnover times, and the quality of organic matter in sediments has been shown to not reflect short-term seasonal inputs of pelagic material in the study area, being mostly dependent on long-term deposition and benthic processes [42]. In addition, as observed for macroalgae and discussed below, the isotopic signatures in our samples strongly resembled those obtained in 2003 [18], confirming the strong stability of their isotopic composition.

The different sediment fractions were obtained by sieving [43]. Phytoplankton and zooplankton were collected with a plankton net (20 micron mesh size), sampling the whole water column to a depth of 100 m, from holes in the sea-ice in spring and in open waters in summer. Since it was composed almost exclusively of copepods, zooplankton was carefully separated from the rest of the bulk sample by hand under a stereoscope. To obtain phytoplankton, the remaining sample was filtered at 100 μm and collected on pre-combusted Whatmann GF/F filters. Sympagic algae were collected by coring the ice-pack at two sites in the inner and outer part of the bay. Algae growing at the interface between ice and water (i.e. up to 2 cm from the bottom of the core, hereafter “interface algae”) were considered separately from those growing within the core (i.e. between 2 cm and 1 m from the bottom, hereafter “core algae”). For both interface and core fractions, microscopic algae (mainly diatoms) were separated from filamentous algae by sieving and filtering on pre-combusted Whatmann GF/F filters.

### Isotopic analyses, niche metrics and Bayesian mixing models

All samples were singly stored at -80°C at the “Mario Zucchelli” Italian Research Station, and were stored at -20°C during transportation to Italy. In Italy, after freeze-drying, all samples were homogenised to a fine powder using a ball mill (Mini-Mill Fritsch Pulverisette 23: Fritsch Instruments, Idar-Oberstein, Germany) [44]. When necessary, samples were pre-acidified (HCl 1 M) to eliminate inorganic carbon, which can interfere with the δ^13^C signature [45]. Un-acidified powder from each sample was also analysed in order to obtain its δ^15^N signature, which is known to be affected by HCl exposure [45]. Samples then underwent SIA by continuous flow mass spectrometer (IsoPrime100, Isoprime Ltd., Cheadle Hulme, UK) coupled with an elemental analyser (Elementar Vario Micro-Cube, Elementar Analysensysteme GmbH, Germany). Each sample was analysed in two replicates, and isotopic signatures were expressed in *δ* units (δ^13^C; δ^15^N) as the per mil (‰) difference with respect to standards: δX (‰) = [(R_sample_—R_standard_)/R_standard_] x 10^3^, where X is ^13^C or ^15^N and R is the corresponding ratio of heavy to light isotopes (^13^C/^12^C or ^15^N/^14^N). The reference materials used were the international Vienna PeeDee Belemnite (PDB) standard for carbon and atmospheric nitrogen (N_2_) for nitrogen. Measurement errors were found to be typically smaller than 0.05‰. For δ^13^C, outputs were corrected for lipid content based on the C/N ratio of each sample [46].

Population-wide niche metrics were applied to *A*. *colbecki* and *S*. *neumayeri* isotopic data in accordance with [37,47,48], using *stable isotope Bayesian ellipses in R* (SIBER) in the R statistical computing package. The bi-dimensional isotopic niche space occupied by the populations was calculated as the total isotopic area (TA) and standard ellipse area (SEAc) (SIBER analysis [47], where “c” stands for ‘‘corrected” by degree of freedom). TA encompasses all specimens and provides the total niche space occupied by each population, while SEAc encompasses the core (around 40%) of the isotopic observations within each population and is poorly sensitive to sample size and isotopic outliers [47,49]. The isotopic dissimilarity between specimens was quantified as the mean isotopic (i.e. Euclidean) distance between each specimen and either its conspecifics (MND*intra*) or its non-conspecifics (MND*inter*) [37]. The higher the MND value, the higher the intra- and inter- specific isotopic dissimilarity respectively.

The diet of *A*. *colbecki* and *S*. *neumayeri* was reconstructed based on the individual isotopic signature of specimens and the season- and depth-specific isotopic signatures of all potential food sources. In order to describe the diet of specimens, a Bayesian mixing model, returning outputs as probability distributions for the parameters of interest, was applied using the mixSIAR package (implemented by R software ver. 2.15.2). The output of the model is a probability density function of plausible values for the proportion of the diet accounted for by each dietary item. A whole-organism trophic enrichment factor (TEF) between consumers and their potential food sources of 0.4±0.2 for carbon and 2.3±0.5 for nitrogen was applied [50]. The isotopic signatures of gut contents were increased by one TEF in order to allow direct comparison between them and the isotopic composition of soft tissues.

Based on the proportional contribution of each food item to the diet of consumers, we calculated the resulting trophic niche width at both the population and individual level as the Shannon diversity (Hs) of resources consumed. Intraspecific diet diversification (π) was calculated as the Bray-Curtis distance between conspecifics based on the identity and the proportion of resources consumed [35,51,52]. Here, the index ranges from 0 (when two specimens consume the same resources and in the same proportion) and 1 (when two specimens consume completely different resources), and can be considered a measure of diet overlap between specimens [37].

Based on the output of Bayesian isotopic mixing models for each specimen, we calculated the uniformity of energy flows between resource guilds and consumers in accordance with [53]. To obtain this, the values for the proportional consumption of individual resource items within each resource guild (i.e. sediment, plankton, benthic and sympagic primary producers) were pooled. The uniformity of energy flows was then calculated as the evenness of interactions (E) between a consumer and the four resource guilds. E varies from 0 (no coupling, when a specimen feeds on one resource guild only) and 1 (complete coupling, when all the four resource guilds contribute equally to the diet of a specimen).

Two-way ANOVA was used to test the effect of time scale (i.e. long- vs. short-term), depth, and their interaction on the consumption of each resource item by consumers. PerMANOVA was used to test for differences in the isotopic composition of both resources and consumers, as well as to test the effect of time scale, depth and their interaction on diet composition.

## Results

### Isotopic niches

Resource guilds differed in their isotopic signatures and were clearly separate in the isotopic niche space (One-way PerMANOVA and post-hoc comparisons, F = 38.1, p< 0.0001), while the mean intraspecific isotopic variability (measured as Euclidean distance) of each resource item between seasons and depths was very low (0.80±0.13‰) (Fig 1 and S1 Table). The isotopic signatures of the red macroalga *P*. *antarctica* were δ^13^C = -37.2±0.3‰ and δ^15^N = 0.1±0.2‰, falling outside the isotopic range occupied by the other resources and by invertebrates.

**Fig 2:**
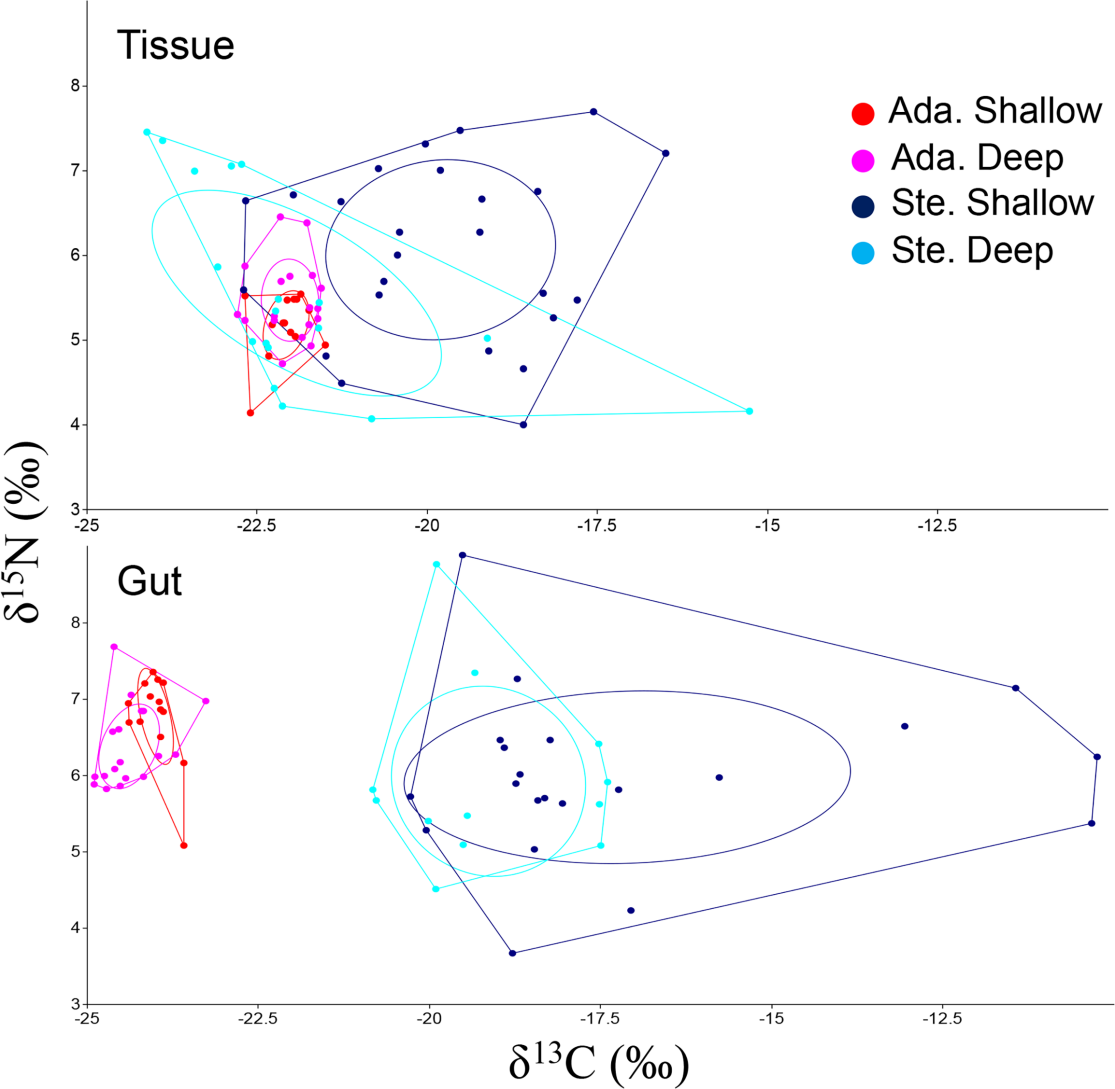
Isotopic niches of consumers. Isotopic distribution of *A*. *colbecki* (ADA) and *S*. *neumayeri* (STE) in shallow (15–25 m depth) and deep (50–150 m depth) waters in the long term (Tissue, based on analysis of soft tissues) and short term (Gut, based on analysis of gut contents). Polygons enclosing isotopic data represent the total isotopic area (TA) occupied by each population, while ellipses are the standard ellipse areas (SEAc) encompassing the core (around 40%) of each population.

Isotopic distribution varied between long-term diet (i.e. considering the isotopic composition of the soft tissues) and short-term diet (i.e. considering the isotopic composition of gut contents) for both *A*. *colbecki* and *S*. *neumayeri*, whereas it varied with depth for *S*. *neumayeri* only (Fig 2; Two-way PerMANOVA, time: F = at least 20.7, p< 0.001 for both species; depth: F = 14.4, p< 0.001 for *S*. *neumayeri*, F = 1.2, p> 0.05 for *A*. *colbecki*; interaction: F = 0.3, p> 0.05 for *S*. *neumayeri*, F = 7.1, p< 0.05 for *A*. *colbecki*). In the long term, *A*. *colbecki* and *S*. *neumayeri* occupied the central part of the isotopic niche space, whereas they had significantly depleted and enriched δ^13^C values respectively in the short term (One-way ANOVA and Tukey’s pairwise comparisons, F = 73.4, p< 0.0001). Accordingly, the *inter*specific isotopic dissimilarity (MND*inter*) was higher in the short term, when no overlap between the SEAcs of the two species was observed, and it was higher in shallow waters (Table 1; Two-way ANOVA, season: F = 79.7, p< 0.0001; depth: F = 10.8, p< 0.01).

**Table 1:** Niche metrics.

	Population			Specimens				
*A*. *colbecki*	TA (‰^2^)	SEAc (‰^2^)	HS*pop*	MND*intra*	MND*inter*	HS*ind*	π	E
Tissue-Shallow	1.04	0.38	1.23	0.58±0.06	2.80±0.08	1.22±0.01	0.07±0.01	0.80±0.01
Tissue-Deep	1.46	0.60	1.47	0.77±0.04	1.76±0.07	1.43±0.02	0.15±0.01	0.92±0.01
Gut-Shallow	0.89	0.40	1.01	0.60±0.03	7.07±0.02	0.93±0.02	0.10±0.01	0.70±0.02
Gut-Deep	1.76	0.69	1.21	0.82±0.06	5.42±0.09	1.19±0.01	0.06±0.00	0.58±0.01
***S*. *neumayeri***								
Tissue-Shallow	14.86	5.39	1.89	2.42±0.10	2.80±0.26	1.81±0.02	0.23±0.01	0.95±0.01
Tissue-Deep	12.10	6.09	1.89	2.54±0.31	1.76±0.66	1.76±0.03	0.26±0.02	0.90±0.03
Gut-Shallow	32.62	10.98	1.53	3.68±0.34	7.07±0.36	1.45±0.03	0.23±0.02	0.91±0.02
Gut-Deep	8.73	5.14	1.49	2.43±0.13	5.42±0.36	1.46±0.07	0.16±0.01	0.86±0.02

*S*. *neumayeri* had a broader isotopic niche than *A*. *colbecki* (Table 1). Accordingly, *intra*specific isotopic dissimilarity (MND*intra*) was higher in the former (Table 1), and it increased linearly with the isotopic niche space, measured as both TA and SEAc (TA: r^2^ = 0.87, p< 0.001; SEAc: r^2^ = 0.98, p< 0.0001). Neither MND*intra* nor MND*inter* values were affected by sample size (*n*) (*n* vs. MND*intra*: r^2^ = 0.18, p = 0.30; *n* vs. MND*inter*: r^2^ = 0.05, p = 0.61).

### Diet composition

**Fig 3:**
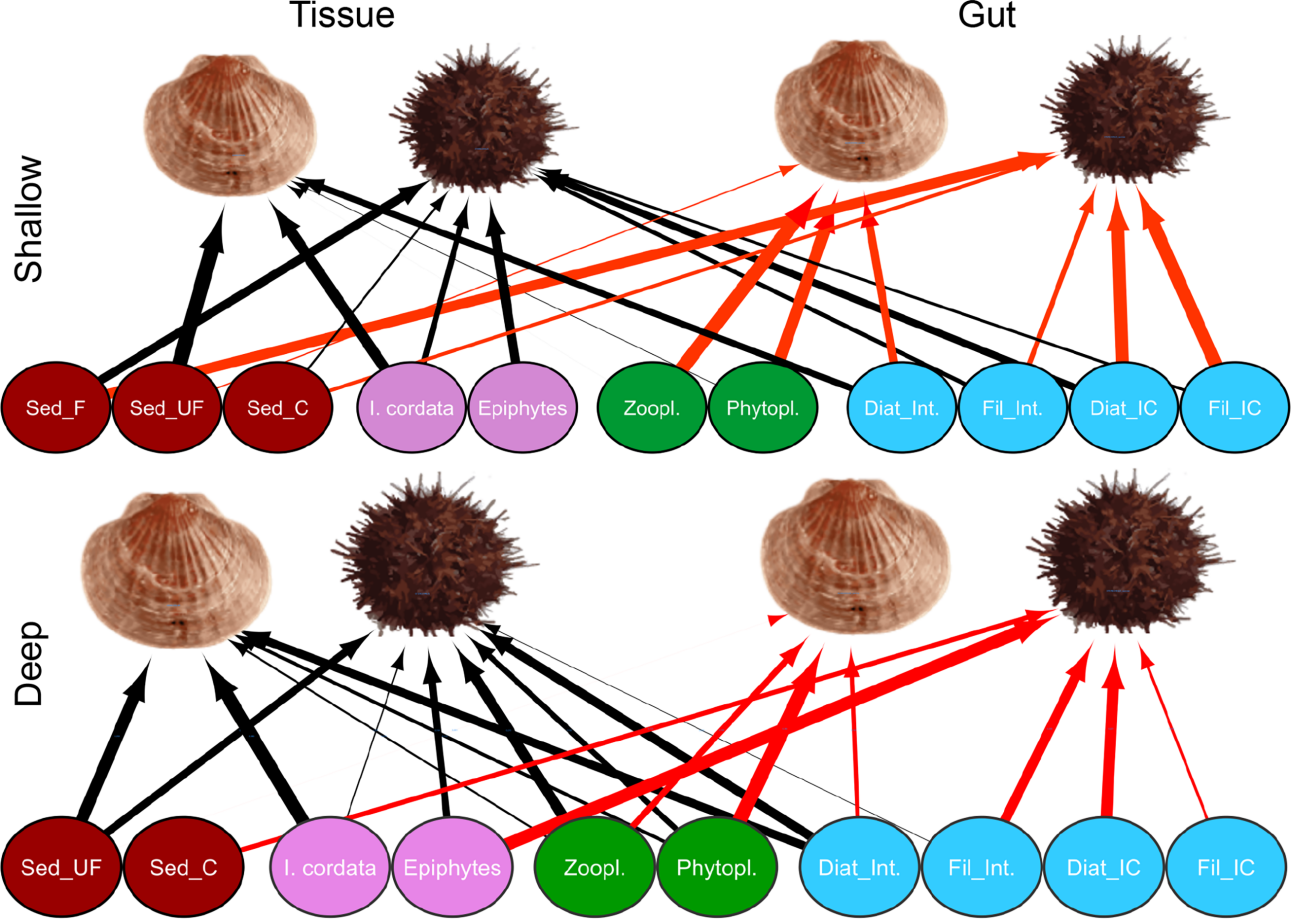
Trophic links between consumers and resources. Resource-consumer food webs depicting the diet composition of the bivalve *A*. *colbecki* and the echinoderm *S*. *neumayeri* in shallow (15–25 m depth) and deep (50–150 m depth) waters in the long term (Tissue: black arrows, based on analysis of soft tissues) and short term (Gut: red arrows, based on analysis of gut contents). Arrow width is proportional to the interaction strength between consumers and resources. Resource guilds are grouped by colour, i.e. brown: organic matter in sediment, including coarse (C), fine (F) and ultra-fine (UF) sediment (Sed.) fractions; purple: benthic primary producers, including the red algae *Iridea cordata* (I. cord.) and epiphytic diatoms (Epi.); green: plankton, including phytoplankton and zooplankton; blue: sympagic algae, including diatoms (Diat.) and filamentous algae (Fil.) growing at the interface between sea-ice and water (Int.) or within the ice core (IC).

According to variations of their isotopic niches, the diet composition of both *A*. *colbecki* and *S*. *neumayeri* varied with time, while it varied with depth for *S*. *neumayeri* only (Fig 3; Two-way PerMANOVA, time: F at least = 171.4, p< 0.001 for both; depth: F at least = 168.2, p< 0.001 for both; interaction: F at least = 81.6, p< 0.001 for both). The number and diversity (Hs) of resources consumed were higher in *S*. *neumayeri* than in *A*. *colbecki*, and for both species they were higher in the long term than in the short term (Table 1 and Fig 3). The contribution of each resource to the diet of both species, as well as diet composition at the individual level, can be found in S3 Fig and S2 Table.

Isotopic and trophic niche metrics of *Adamussium colbecki* and *Sterechinus neumayeri* at each depth (shallow: 15–25 m; deep: 50–150 m) and in the long term (Tissue, based on analysis of soft tissues) and short term (Gut, based on analysis of gut contents). TA: total isotopic niche area; SEAc: standard ellipse area corrected for degrees of freedom. MND*intra* and MND*inter*: intra- and inter-specific mean isotopic distance between specimens respectively. Hs: trophic niche width calculated as the Shannon diversity of resources consumed at both population (*pop*) and individual (*ind*) level. π: degree of intraspecific diet differentiation (see materials and methods). E: degree of coupling of the energy channels (sediment, benthic, pelagic, and sympagic producers) in the food web, ranging from 0 (consumers feeding on one channel only) to 1 (consumers equally feeding on all channels).

#### Diet of *Adamussium colbecki*

In the long term, *A*. *colbecki* relied mainly on the ultra-fine sediment fraction and *I*. *cordata*, while *P*. *antarctica* was not consumed. Consumption of phytoplankton and zooplankton was limited, their contribution being slightly but significantly higher in deep waters (Fig 3; Two-way ANOVA, season: F = at least 464.3, p< 0.0001 for both; depth: F = at least 207.4, p< 0.0001 for both; interaction: F = at least 325.0, p< 0.0001 for both). The consumption of sympagic interface diatoms did not vary over time, and was higher in shallow waters (Two-way ANOVA, season: F = 1.6, p = 0.21; depth: F = 23.20, p< 0.0001; interaction: F = 34.2, p< 0.0001). In the short term, *I*. *cordata* was not consumed and consumption of ultra-fine sediment was lower (Two-way ANOVA, season: F = 808.9, p< 0.0001; depth: F = 177.2, p< 0.0001; interaction: F = 121.2, p< 0.0001), while that of phytoplankton and zooplankton was higher (Fig 3 and S2 Table).

#### Diet of *Sterechinus neumayeri*

All fractions of sediment were consumed by *S*. *neumayeri* in the long term, mostly in shallow waters. In the short term, consumption of coarse sediment was higher whereas ultra-fine sediment was not consumed (Fig 3 and S2 Table; Two-way ANOVA; season, F = 1.2, p> 0.05; depth, F = 150.3, p< 0.0001; interaction, F = 0.6, p> 0.05 for the fine fraction; season, F = 61.5, p< 0.0001; depth: F = 37.5, p< 0.0001; interaction: F = 50.5, p< 0.0001 for the coarse fraction). Among the red algae, *P*. *antarctica* was not consumed and *I*. *cordata* was consumed in the long term only, mostly in shallow waters (Two way ANOVA, season, F = 140.0, p< 0.0001; depth, F = 8.1, p< 0.01; interaction, F = 9.4, p< 0.01). Phytoplankton and zooplankton were also consumed in the long term only, but in deep waters. The overall contribution of sympagic algae to diet was higher in the short term, and higher in shallow waters (Two way ANOVA, season, F = 124.3, p< 0.0001; depth, F = 43.4, p< 0.0001; interaction, F = 0.7, p> 0.05), while epiphytic diatoms were mostly consumed in deep waters in the short term (Two way ANOVA, season, F = 12.6, p< 0.001; depth, F = 65.5, p< 0.0001; interaction, F = 154.9, p< 0.0001).

#### Individual diets and degree of coupling of energy channels in the food web

For both species, the individual trophic niche width (i.e. the diversity of resources consumed by each specimen, Hs*ind*) was greater in the long term, and for *A*. *colbecki* alone it was greater in deep waters (Table 1; Two-way ANOVA, *A*. *colbecki*, season: F = 260.4, p< 0.0001; depth: F = 215.4, p< 0.0001; interaction: F = 8.6, p< 0.01. *S*. *neumayeri*, season: F = 147.9, p< 0.0001; depth: F = 0.1, p> 0.05; interaction: F = 1.1, p> 0.05). Intraspecific diet diversification (π) was higher in *S*. *neumayeri* than in *A*. *colbecki* (Table 1). For both species, it was higher in the long than in the short term in deep waters. (Table 1; Two-Way ANOVAs, *A*. *colbecki*, season: F = 20.9, p< 0.0001; depth: F = 82.9, p< 0.0001: interaction: F = 9.0, p< 0.01. *S*. *neumayeri*, season: F = 6.8, p = 0.01; depth: F = 0.5, p> 0.05; interaction: F = 12.5, p< 0.001). The coupling of energy channels within the food web (E) was more marked in the long than in the short term, and more for *S*. *neumayeri* than for *A*. *colbecki* (Table 1; Two-Way ANOVA, species: F = 143.5, p< 0.0001; season: F = 102.8, p< 0.0001; interaction: F = 58.8, p< 0.0001), increasing with the trophic niche width of specimens (y = 0.34x + 0.35, r^2^ = 0.60, p< 0.05).

### Body size and diet

For both species, the mean size of sampled specimens did not vary with depth (*A*. *colbecki*: t = 1.19, p> 0.05; *S*. *neumayeri*: t = 0.92, p> 0.05). The distribution of sizes matched criteria for normality, implying that intermediate-size specimens were the most abundant (Variance: Shapiro-Wilk test, p normal> 0.05 for both species; skewness: Jarque Bera test, p normal> 0.05 for both species).

**Fig 4:**
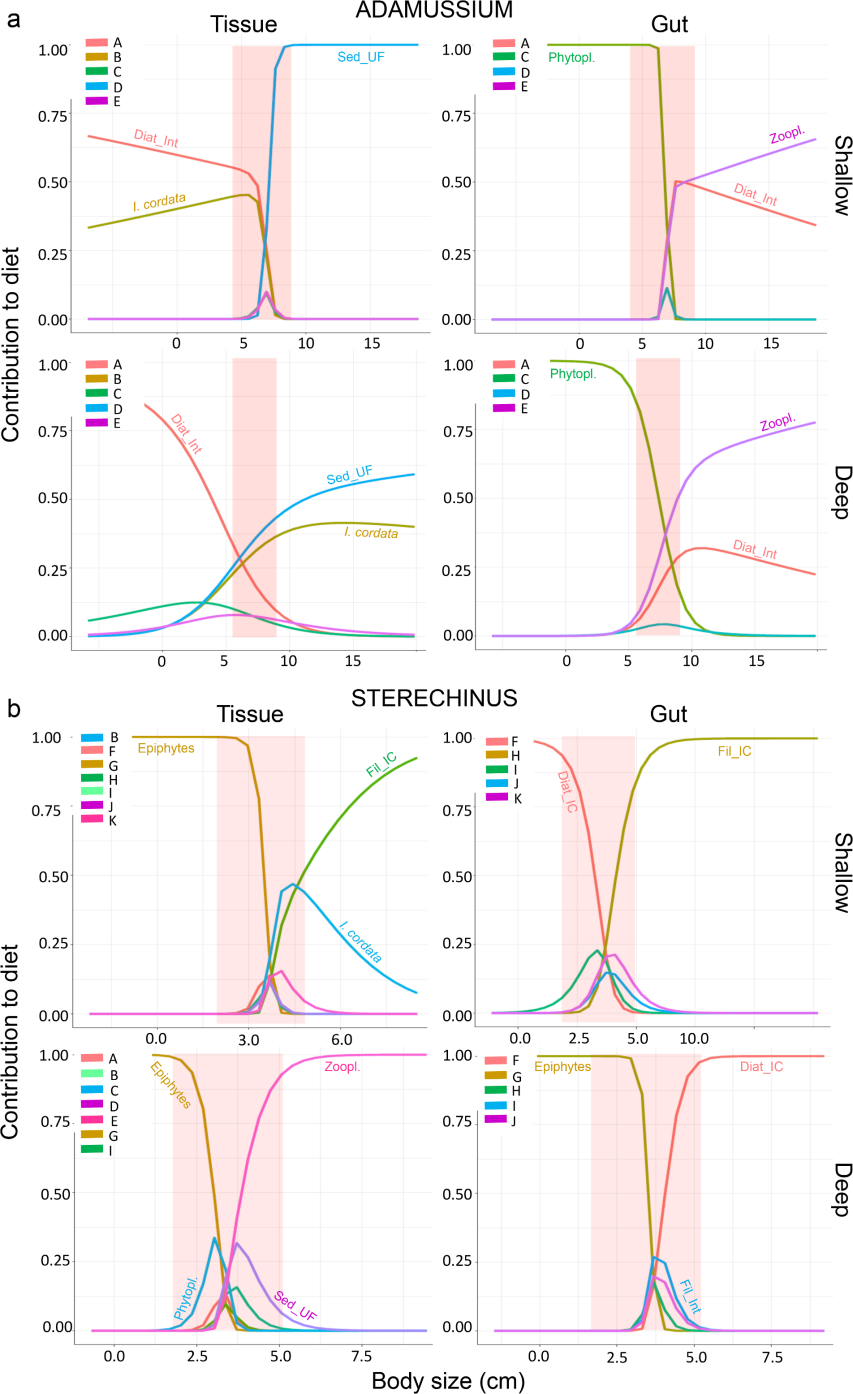
Body size and diet. Dietary change with specimen size in shallow (15–25 m depth) and deep (50–150 m depth) waters in the long term (Tissue, based on analysis of soft tissues) and short term (Gut, based on analysis of gut contents) (mixSIAR, R software, version 2.15.2). (a) = *A*. *colbecki*; (b) = *S*. *neumayeri*. The software predicts the contribution of resources over a wider size range than that of the original samples; the red shaded areas indicate the size range actually observed for the specimens analysed (note differences on the horizontal axes between panels). Each coloured line indicates a given resource. A: interface sympagic diatoms, B: *I*. *cordata*, C: phytoplankton, D: ultra-fine sediment, E: zooplankton, F: ice-core diatoms, G: epiphytic benthic diatoms, H: ice-core filamentous sympagic algae, I: interface filamentous sympagic algae, J: coarse sediment, K: fine sediment.

Intermediate-size specimens were generally found to consume a greater diversity of resources with respect to small and large organisms (Fig 4). For *A*. *colbecki*, sympagic diatoms were preferred by smaller specimens while sediment was preferred by larger ones in the long term (Fig 4A). In the short term, small and large specimens specialised on phytoplankton and zooplankton respectively. Epiphytic diatoms were generally preferred by small specimens of *S*. *neumayeri* in both the long and the short term, whereas large individuals specialised on various food items depending on the time scale and depth (Fig 4B).

## Discussion

This study provides quantitative information on time- and depth-wise changes in the isotopic and trophic niche structure of two key Antarctic consumers (*A*. *colbecki* and *S*. *neumayeri*) and associated consequences for nutrient transfer within the food web. The separate analysis of sediment fractions and the sympagic algal community differing in both size (i.e. diatoms vs. filamentous algae) and association with sea-ice (i.e. core vs. interface algae) made it possible to identify variations in consumer diets, which would not have been observed otherwise. The isotopic signatures of basal resources clearly distinguished sympagic from pelagic and benthic producers, and were highly conserved across both seasons and depths, reflecting those obtained in 2003 [18] at the same study site. While the 2003 study [18] did not distinguish between sediment fractions, their values differ by only 0.3‰ and 0.9‰ from the mean δ^13^C and δ^15^N values of our sediment samples respectively. Similarly, the current and 2003 δ^13^C values of both benthic and sympagic algae were highly similar, including the highly ^13^C depleted *P*. *antarctica*, confirming the conserved isotopic composition of resources over many years. The isotopic similarity of resources sampled in both spring and summer in the current study suggests that this also holds on the seasonal time scale. Significant differences in the isotopic distribution of both *A*. *colbecki* and *S*. *neumayeri*, as well as the absence of isotopic overlap of the SEAC of each species between the long and the short term (with the exception of *S*. *neumayeri* in shallow waters, where its SEAc was broader in the short than in the long term), enable us to state with confidence that changes in isotopic niches actually reflect a shift in the diet of specimens over time. We acknowledge that differences in tissue turnover rates and longevities could affect the interpretation of isotopic data as a proxy for food selection over time. Nevertheless, given the slow metabolic rates of Antarctic organisms, such differences are not expected to unduly affect our comparison based on gut contents and soft tissues. Such long- vs. short-term diet comparison is of ecological relevance for the study system. Indeed, the summer fallout of freshly produced sympagic and pelagic material is expected to occur over a short time period in the study area [12,13,54], and the feeding activity of benthic Antarctic organisms is expected to rapidly increase accordingly [55, 56].

In the short term, *A*. *colbecki* and *S*. *neumayeri* specialised on plankton and sympagic algae respectively, the availability of which is expected to increase rapidly during and soon after seasonal sea-ice melting [12,13,54]. Trophic specialisation can occur as a consequence of an increase in resource availability [29,37]. The increased productivity in summer could thus explain the narrower trophic niches of specimens. Although they did not represent the principal food items, pelagic and sympagic production also contributed to the nutrients assimilated by both species in the long term. In spring, after the activation of photosynthesis, transportation via advection from the open waters of the Terra Nova Bay polynya may represent a food input before local sea-ice break-up and the release of in-situ production [18,57]. Regardless of the time scale, the consumption of sympagic algae in shallow waters was greater than in deep waters, while that of plankton was lower, confirming the need for a better understanding of food web organisation along depth gradients. Concerning the depths considered in this study, iceberg scouring is known to shape benthos composition and abundance in shallow waters, while the sea floor in the deep waters of the study area hosts higher diversity and a more clearly defined community composition, as is generally expected in Antarctic coastal communities [58,59]. Although sampling in deep waters encompassed a wider depth range than in shallow waters, the intraspecific isotopic variability of both species was similar between depths, being even higher in shallow than in deep waters for *S*. *neumayeri*, thus excluding a bias arising from different depth sampling ranges.

Quantifying the individual importance of distinct energy channels in polar food webs is central to understanding how these ecosystems function [8]. Here, changes in the importance of pelagic, sympagic, benthic and sediment energy channels and the degree of coupling between them in the food web were a direct consequence of modifications in the trophic niches of specimens, which were broader in the long term, when consumers foraged more evenly across the four energy channels supporting the food web. Feeding by consumers across energy channels differing in turnover rate and productivity stabilises food webs [7]. Thus, our results point to an important role of generalist consumers in the stability of nutrient transfer across trophic levels in the long term, before the sea-ice broke up. While the consumption of sympagic and pelagic production increased in the short term, nutrient uptake from benthic producers and sediment, whose availability is little affected by season, decreased. While belonging to different feeding guilds, facilitation between the two species can be hypothesised in the long term. Indeed, the consumption of fresh algal debris by *A*. *colbecki* is expected to occur as a consequence of grazing activity by *S*. *neumayeri* and other herbivore species, which makes algal fragments accessible to the filter feeder following deposition in sediments or drifting in the water column. In parallel, *A*. *colbecki* can process up to 14% of pelagic productivity in the study area, recycling into sediments around 65% of ingested food via biodeposition [32].

Body size played a key role in explaining intraspecific differences in the diet of specimens. Although it is a key factor structuring populations and natural communities [60–62], little is known about the role of body size in polar benthic food webs. The data presented here provide unprecedented insight into size-related changes in resource preference in Antarctic benthic species. Among other observations, epiphytic diatoms were found to play a dominant role in the diet of small specimens of *S*. *neumayeri*. Despite their importance in food webs, benthic epiphytic diatoms have been studied to a much lesser extent than their planktonic and sympagic counterparts in Antarctica [41]. Here, as observed in coastal benthic systems at lower latitudes [63,64], while *P*. *antarctica* was not directly consumed, it provided a key substrate for epiphytic colonisation and foraging by consumers.

### Concluding remarks

Polar benthic species’ growth and reproduction are mainly limited by food [56,57]. In turn, the availability of sympagic algae to benthic consumers is closely related to sea-ice dynamics, which also affects the timing and magnitude of summer phytoplankton blooms in coastal waters [12,15,16]. Thus, potential climate-driven variations in the timing and quantity of nutrient inputs to consumers may have profound implications for the stability of food webs and the persistence of their constituent species, which have adapted their trophic niches to a highly predictable schedule of resource inputs [55,56]. Here, we focused on two key Antarctic species that are widely distributed and often found in association along Antarctic coasts. The isotopic approach was effective in describing intra- and inter-specific changes in resource use and nutrient transfer across trophic levels associated with the higher availability of resources expected in summer. The observation of foraging optimisation in Antarctic taxa, in conjunction with the study of diet modification with body size, will help to apply classical ecological theories concerning niche partitioning by species along resource gradients to Antarctic communities [28,29], thereby improving our understanding of their structure and functioning. In this perspective, laboratory measurements of the turnover rates of consumer tissues will improve accuracy in quantifying temporal changes in species’ diets and links in the food web based on the isotopic analysis of Antarctic taxa.

## Supporting information

S1 Fig Distribution maps. Occurrence of (a) *Sterechinus neumayeri* and (b) *Adamussium colbecki* along Antarctic coasts. Distribution maps are created from the World Register of Marine Species (WoRMS, www.marinespecies.org).
(TIF)

S2 Fig Study area. The two sampling transects in the Tethys Bay, Ross Sea, are shown in red. D: deep waters (50–150 m depth); S: shallow waters (15–25 m depth). The yellow dashed lines indicate the position of the sea-ice margin six and thirteen days before our sampling. MZS: the Italian research station “Mario Zucchelli”.
(TIF)

S3 Fig Individual trophic niches. Radar charts displaying the proportional contribution of resources to the long-term (Tissue, based on analysis of soft tissues) and short-term (Gut, based on analysis of gut contents) diets of *A*. *colbecki* (Adamussium, left charts) and *S*. *neumayeri* (Sterechinus, right charts) in shallow (15–25 m) and deep (50–150 m) waters. Each axis of the chart represents one trophic niche axis (i.e. one resource item). Each grey line represents the trophic niche of one specimen, while the red line represents the mean diet at the population level (software: mixSIAR package, R version 2.15.2). Each tick-mark on the resource axes represents a contribution of 0.1 (10%) of that resource to the diet of consumers. Please note differences between charts in terms of number of tick-marks on axes. For resource abbreviations, please refer to S2 Table.
(PDF)

S1 Table δ^13^C and δ^15^N of food sources. Isotopic signatures (mean±S.D.) of resources available to reconstruct the diet of benthic consumers at Tethys Bay (Ross Sea) in shallow (15–25 m depth) and deep (50–150 m depth) waters collected both in spring (i.e. November) and summer (i.e. January). In some cases, values are repeated between seasons or depths if specific samples were not available (see the material and methods section for details). INT: diatoms (Diat.) or filamentous (Fil.) sympagic algae growing at the interface between sea water and sea ice (up to 2 cm within the ice-core). IC: diatoms or filamentous sympagic algae growing from 2 cm up to 1 m within the ice core. Sed_UF, _F and _C refer to the ultra-fine, fine and coarse fractions of organic matter in sediments respectively.
(PDF)

S2 Table Contribution of resources to the diet of consumers. Mean, minimum and maximum proportional contribution of each resource to the long-term (Tissue, based on analysis of soft tissues) and short-term (Gut, based on analysis of gut contents) diets of *Adamussium colbecki* (ADAMUSSIUM) and *Sterechinus neumayeri* (STERECHINUS) in shallow (15–25 m depth) and deep (50–150 m depth) waters at Tethys Bay, Ross Sea (software: mixSIAR package, R version 2.15.2). Mean, min and max refer to the contribution of each resource across specimens. INT: diatoms (Diat.) or filamentous (Fil.) sympagic algae growing at the interface between sea water and sea-ice (up to 2 cm within the ice-core). IC: diatoms or filamentous sympagic algae growing from 2 cm up to 1 m within the ice core. Sed_UF, _F and _C refer to the ultra-fine, fine and coarse fractions of organic matter in sediments respectively.
(PDF)
