# Correction: Time- and depth-wise trophic niche shifts in Antarctic benthos

**DOI:** 10.1371/journal.pone.0197009

**Published:** 2018-05-02

**Authors:** The PLOS ONE Staff

The legend for Table 1 appears incorrectly in the Results section of the paper. Please see the correct version of Table 1 here.

**Table 1:** Niche metrics.

	Population			Specimens				
*A*. *colbecki*	TA (‰^2^)	SEAc (‰^2^)	HS*pop*	MND*intra*	MND*inter*	HS*ind*	π	E
Tissue-Shallow	1.04	0.38	1.23	0.58±0.06	2.80±0.08	1.22±0.01	0.07±0.01	0.80±0.01
Tissue-Deep	1.46	0.60	1.47	0.77±0.04	1.76±0.07	1.43±0.02	0.15±0.01	0.92±0.01
Gut-Shallow	0.89	0.40	1.01	0.60±0.03	7.07±0.02	0.93±0.02	0.10±0.01	0.70±0.02
Gut-Deep	1.76	0.69	1.21	0.82±0.06	5.42±0.09	1.19±0.01	0.06±0.00	0.58±0.01
***S*. *neumayeri***								
Tissue-Shallow	14.86	5.39	1.89	2.42±0.10	2.80±0.26	1.81±0.02	0.23±0.01	0.95±0.01
Tissue-Deep	12.10	6.09	1.89	2.54±0.31	1.76±0.66	1.76±0.03	0.26±0.02	0.90±0.03
Gut-Shallow	32.62	10.98	1.53	3.68±0.34	7.07±0.36	1.45±0.03	0.23±0.02	0.91±0.02
Gut-Deep	8.73	5.14	1.49	2.43±0.13	5.42±0.36	1.46±0.07	0.16±0.01	0.86±0.02

Isotopic and trophic niche metrics of *Adamussium colbecki* and *Sterechinus neumayeri* at each depth (shallow: 15–25 m; deep: 50–150 m) and in the long term (Tissue, based on analysis of soft tissues) and short term (Gut, based on analysis of gut contents). TA: total isotopic niche area; SEAc: standard ellipse area corrected for degrees of freedom. MND*intra* and MND*inter*: intra- and inter-specific mean isotopic distance between specimens respectively. Hs: trophic niche width calculated as the Shannon diversity of resources consumed at both population (*pop*) and individual (*ind*) level. π: degree of intraspecific diet differentiation (see materials and methods). E: degree of coupling of the energy channels (sediment, benthic, pelagic, and sympagic producers) in the food web, ranging from 0 (consumers feeding on one channel only) to 1 (consumers equally feeding on all channels).
