# Supplementary figures and images for: Time- and depth-wise trophic niche shifts in Antarctic benthos
Source: PLoS One. 2018 Mar 23;13(3):e0194796. doi: 10.1371/journal.pone.0194796 (PMC5865725; doi:10.1371/journal.pone.0194796)

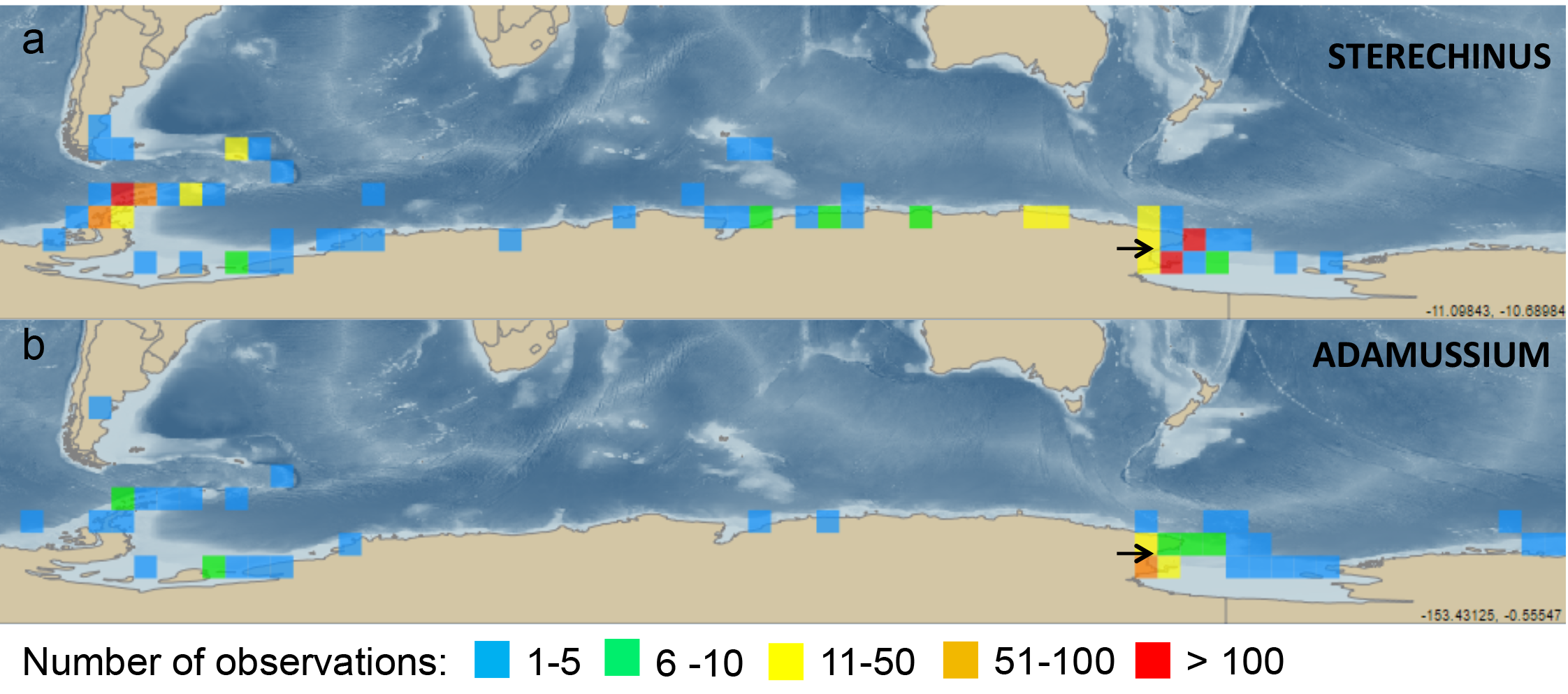

Supplement: S1 Fig — Occurrence of (a) Sterechinus neumayeri and (b) Adamussium colbecki along Antarctic coasts. Distribution maps are created from the World Register of Marine Species (WoRMS, www.marinespecies.org). (TIF) [file pone.0194796.s001.tif]

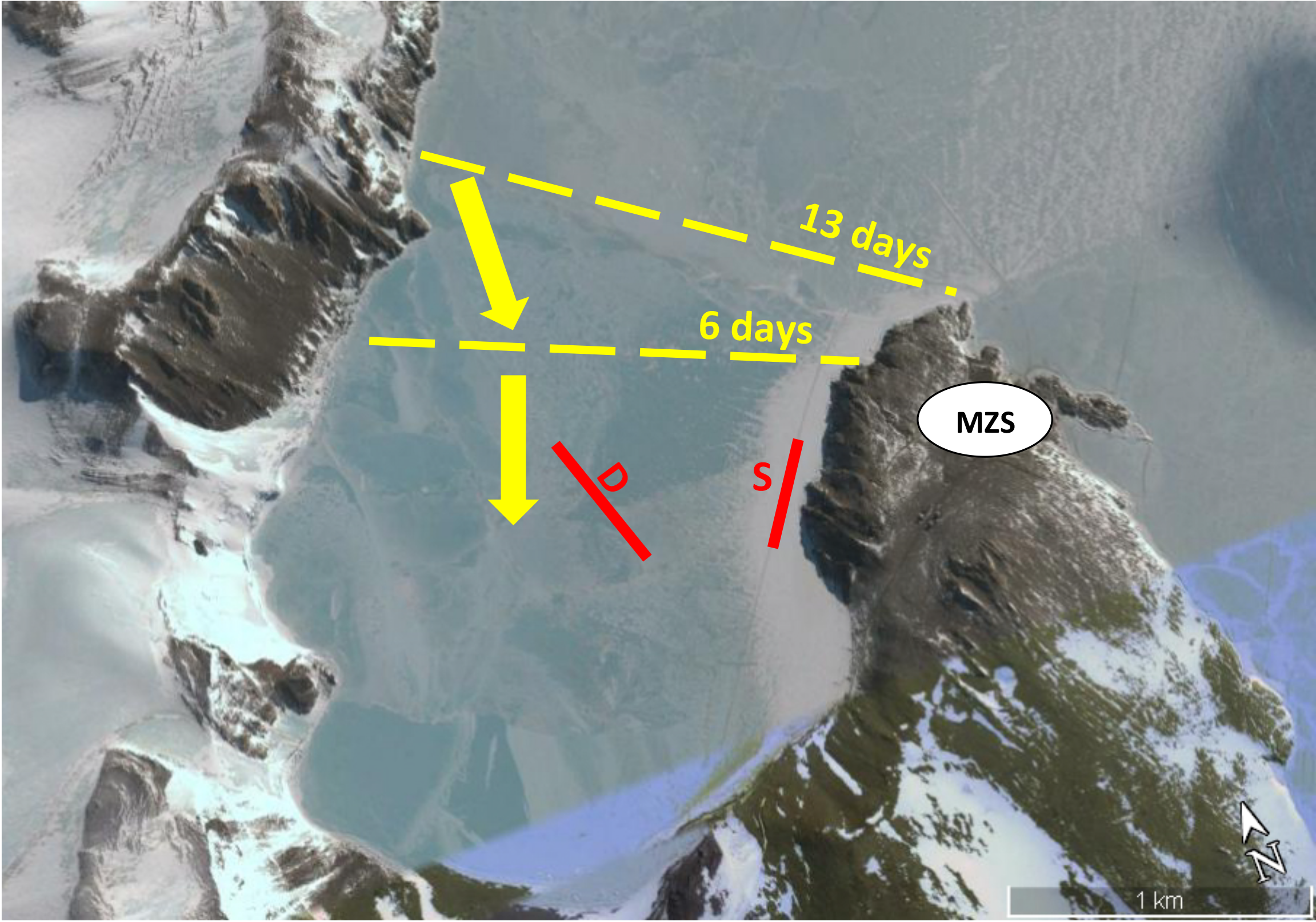

Supplement: S2 Fig — The two sampling transects in the Tethys Bay, Ross Sea, are shown in red. D: deep waters (50–150 m depth); S: shallow waters (15–25 m depth). The yellow dashed lines indicate the position of the sea-ice margin six and thirteen days before our sampling. MZS: the Italian research station “Mario Zucchelli”. (TIF) [file pone.0194796.s002.tif]

# ADAMUSSIUM

# STERECHINUS

## TISSUE-SHALLOW

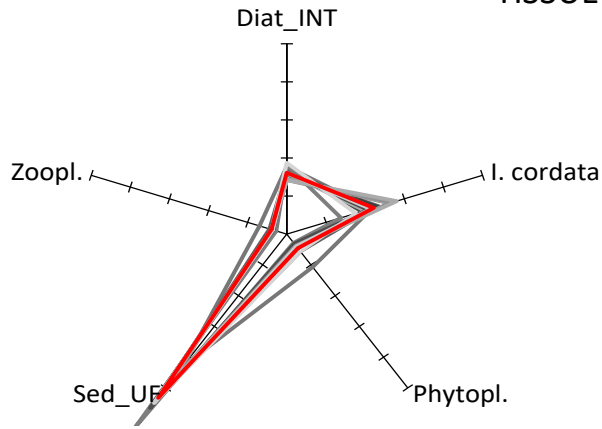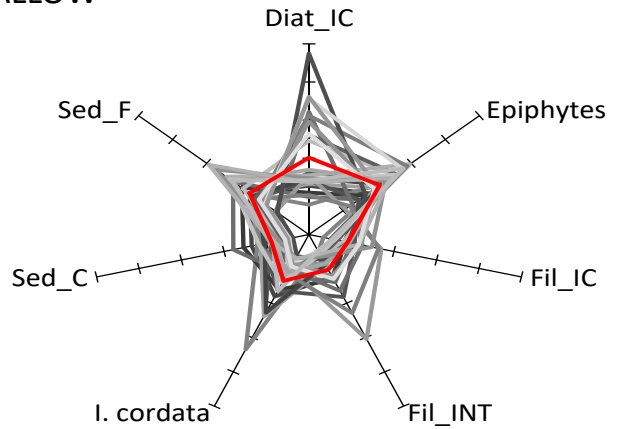

## TISSUE-DEEP

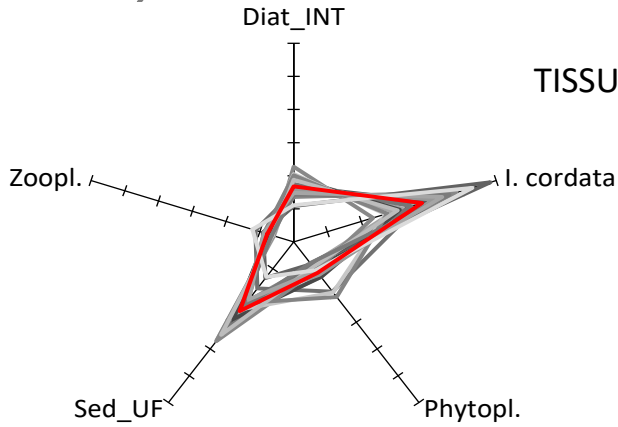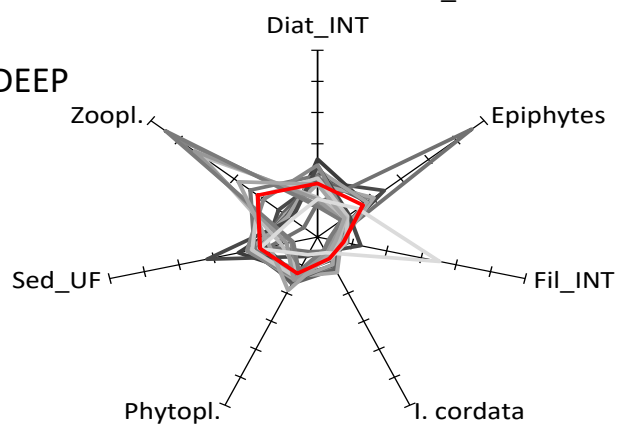

## GUT-SHALLOW

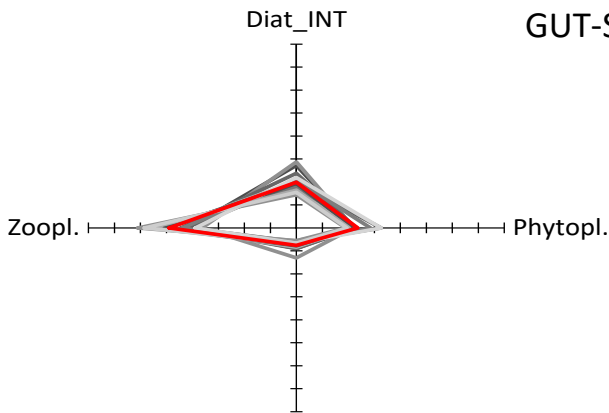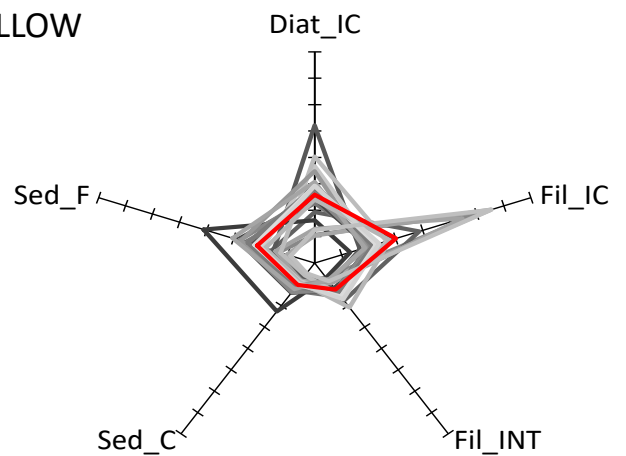

## GUT-DEEP

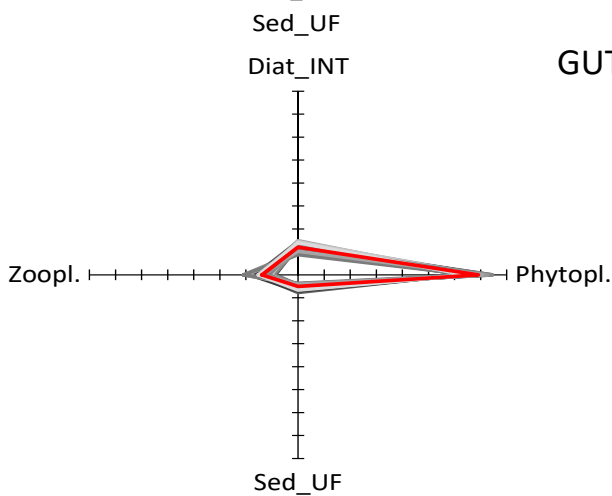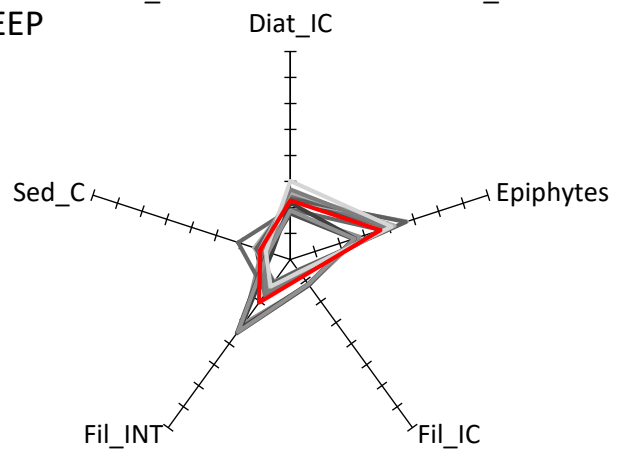

Supplement: S3 Fig — Radar charts displaying the proportional contribution of resources to the long-term (Tissue, based on analysis of soft tissues) and short-term (Gut, based on analysis of gut contents) diets of A. colbecki (Adamussium, left charts) and S. neumayeri (Sterechinus, right charts) in shallow (15–25 m) and deep (50–150 m) waters. Each axis of the chart represents one trophic niche axis (i.e. one resource item). Each grey line represents the trophic niche of one specimen, while the red line represents the mean diet at the population level (software: mixSIAR package, R version 2.15.2). Each tick-mark on the resource axes represents a contribution of 0.1 (10%) of that resource to the diet of consumers. Please note differences between charts in terms of number of tick-marks on axes. For resource abbreviations, please refer to S2 Table. (PDF) [file pone.0194796.s003.pdf]
