# Supplementary material for: Time- and depth-wise trophic niche shifts in Antarctic benthos
Source: PLoS One. 2018 Mar 23;13(3):e0194796. doi: 10.1371/journal.pone.0194796 (PMC5865725; doi:10.1371/journal.pone.0194796)
Supplement: S1 Table — Isotopic signatures (mean±S.D.) of resources available to reconstruct the diet of benthic consumers at Tethys Bay (Ross Sea) in shallow (15–25 m depth) and deep (50–150 m depth) waters collected both in spring (i.e. November) and summer (i.e. January). In some cases, values are repeated between seasons or depths if specific samples were not available (see the material and methods section for details). INT: diatoms (Diat.) or filamentous (Fil.) sympagic algae growing at the interface between sea water and sea ice (up to 2 cm within the ice-core). IC: diatoms or filamentous sympagic algae growing from 2 cm up to 1 m within the ice core. Sed_UF, _F and _C refer to the ultra-fine, fine and coarse fractions of organic matter in sediments respectively. (PDF) [file pone.0194796.s004.pdf]

S1 Table. δ^{13}C and δ^{15}N of food sources. Isotopic signatures (mean±S.D.) of resources available to benthic consumers at Tethys Bay (Ross Sea) in shallow (15-25) and deep (50-150) waters collected both in spring (i.e. November, before seasonal sea-ice break up) and summer (i.e. January, few days after seasonal sea-ice break up), and considered for the diet reconstruction by means of Bayesian mixing models. In some cases, values are repeated between seasons or depths if specific samples were not available (see the material and methods section for details). INT: diatoms (Diat.) or filamentous (Fil.) sympagic algae growing at the interface between sea water and sea ice (up to 2cm within the ice-core). IC: diatoms or filamentous sympagic algae growing from 2cm up to 1m within the ice core. Sed_UF, _F, and _C refer to the ultra-fine, fine and coarse fractions of organic matter in sediments respectively. The intraspecific isotopic variability (measured as Euclidean distance) of each resource item between seasons and depths was very low (0.80±0.13‰), and it was significantly lower than the mean Euclidean distance between different resource items, in both seasons and at both depths (7.22±0.66‰, T-test, t = 9.5, p< 0.0001, including *P. antarctica*, which had very ^{13}C depleted values; 5.23±0.38‰, T-test, t = 10.9, p< 0.0001, excluding *P. antarctica*).

	SPRING				SUMMER			
	SHALLOW		DEEP		SHALLOW		DEEP	
RESOURCE	δ13C (‰)	δ15N (‰)	δ13C (‰)	δ15N (‰)	δ13C (‰)	δ15N (‰)	δ13C (‰)	δ15N (‰)
P. antarctica	-37.7±0.3	0.6±1.4	-36.7±1.0	-0.3±0.2	-37.3±1.3	0.0±0.3	-36.7±1.0	-0.3±0.2
I. cordata	-22.1±0.1	4.3±0.1	-21.4±1.4	4.2±0.2	-21.6±0.1	4.4±0.1	-21.4±1.4	4.2±0.2
Epiphytes	-21.8±0.8	4.1±1.1	-20.9±1.6	3.8±1.3	-21.4±0.8	4.3±1.2	-20.9±1.6	3.8±1.3
Phytoplankton	-24.2±0.2	3.6±0.3	-24.1±1.0	4.7±0.7	-24.1±1.7	4.7±0.7	-24.1±1.0	4.7±0.7
Zooplankton	-24.0±0.9	6.1±1.0	-24.1±0.2	6.4±0.4	-24.1±0.1	6.4±0.4	-24.1±0.2	6.4±0.4
Sed_UF	-22.1±0.6	1.9±0.9	-21.2±1.0	0.0±0.5	-22.1±0.6	1.9±0.9	-21.2±1.0	0.0±0.5
Sed_F	-21.3±0.9	5.8±1.1	-20.8±0.3	5.3±1.1	-21.3±0.9	5.8±1.1	-20.8±0.3	5.3±1.1
Sed_C	-22.1±0.2	7.0±0.6	-22.2±1.0	7.5±0.5	-22.1±0.2	7.0±0.6	-22.2±1.0	7.5±0.5
Diat_IC	-18.8±1.8	0.8±1.2	-18.8±1.8	0.8±1.2	-18.8±1.8	0.8±1.2	-18.8±1.8	0.8±1.2
Diat_INT	-22.8±1.1	0.9±1.3	-22.8±1.1	0.9±1.3	-22.8±1.1	0.9±1.3	-22.8±1.1	0.9±1.3
Fil_IC	-12.4±1.9	2.6±1.1	-12.4±1.9	2.6±1.1	-12.4±1.9	2.6±1.1	-12.4±1.9	2.6±1.1
Fil_INT	-16.0±0.1	2.9±0.5	-16.0±0.1	2.9±0.5	-16.0±0.1	2.9±0.5	-16.0±0.1	2.9±0.5
