# Supplementary material for: Time- and depth-wise trophic niche shifts in Antarctic benthos
Source: PLoS One. 2018 Mar 23;13(3):e0194796. doi: 10.1371/journal.pone.0194796 (PMC5865725; doi:10.1371/journal.pone.0194796)
Supplement: S2 Table — Mean, minimum and maximum proportional contribution of each resource to the long-term (Tissue, based on analysis of soft tissues) and short-term (Gut, based on analysis of gut contents) diets of Adamussium colbecki (ADAMUSSIUM) and Sterechinus neumayeri (STERECHINUS) in shallow (15–25 m depth) and deep (50–150 m depth) waters at Tethys Bay, Ross Sea (software: mixSIAR package, R version 2.15.2). Mean, min and max refer to the contribution of each resource across specimens. INT: diatoms (Diat.) or filamentous (Fil.) sympagic algae growing at the interface between sea water and sea-ice (up to 2 cm within the ice-core). IC: diatoms or filamentous sympagic algae growing from 2 cm up to 1 m within the ice core. Sed_UF, _F and _C refer to the ultra-fine, fine and coarse fractions of organic matter in sediments respectively. (PDF) [file pone.0194796.s005.pdf]

S2 Table. Contribution of resources to the diet of consumers. Mean, minimum and maximum proportional contribution of each resource to the long (by considering isotopic signatures in soft tissues) and short (by considering isotopic signatures in gut content) term diets of *Adamussium colbecki* (ADAMUSSIUM) and *Sterechinus neumayeri* (STERECHINUS) in shallow (15-25m depth) and deep (50-150m depth) waters at Tethys Bay, Ross Sea (software: mixSIAR package, R version 2.15.2). Mean, min and max refer to the contribution of each resource across specimens. INT: diatoms (Diat.) or filamentous (Fil.) sympagic algae growing at the interface between sea water and sea-ice (up to 2cm within the ice-core). IC: diatoms or filamentous sympagic algae growing from 2cm up to 1m within the ice core. Sed_UF, _F, and _C refer to the ultra-fine, fine and coarse fractions of organic matter in sediments respectively.

LONG TERM

Species	Depth	Resoruce	Mean	Min	Max
ADAMUSSIUM	SHALLOW	Diat_INT	0.16	0.14	0.19
		I. cordata	0.22	0.14	0.28
		Phytopl.	0.05	0.03	0.11
		Sed_UF	0.53	0.43	0.63
		Zoopl.	0.04	0.03	0.07
	DEEP	Diat_INT	0.17	0.11	0.23
		I. cordata	0.38	0.24	0.58
		Phytopl.	0.12	0.08	0.21
		Sed_UF	0.26	0.13	0.37
		Zoopl.	0.08	0.06	0.12
STERECHINUS	SHALLOW	Diat_IC	0.20	0.08	0.48
		Epiphytes	0.21	0.11	0.30
		Fil_IC	0.09	0.03	0.17
		Fil_INT	0.10	0.05	0.30
		I. cordata	0.14	0.06	0.34
		Sed_C	0.08	0.04	0.18
		Sed_F	0.18	0.09	0.29
	DEEP	Diat_INT	0.17	0.10	0.25
		Epiphytes	0.16	0.09	0.56
		Fil_INT	0.08	0.03	0.35
		I. cordata	0.08	0.04	0.13
		Phytopl.	0.13	0.05	0.19
		Sed_UF	0.17	0.07	0.32
		Zoopl.	0.22	0.05	0.55

SHORT TERM

Species	Depth	Resoruce	Mean	Min	Max
ADAMUSSIUM	SHALLOW	Diat_INT	0.20	0.14	0.29
		Phytopl.	0.24	0.19	0.33
		Sed_UF	0.08	0.06	0.13
		Zoopl.	0.49	0.36	0.61
	DEEP	Diat_INT	0.12	0.09	0.15
		Phytopl.	0.69	0.62	0.74
		Sed_UF	0.05	0.04	0.08
		Zoopl.	0.14	0.09	0.21
STERECHINUS	SHALLOW	Diat_IC	0.26	0.10	0.52
		Fil_IC	0.30	0.13	0.65
		Fil_INT	0.12	0.07	0.21
		Sed_C	0.10	0.05	0.23
		Sed_F	0.21	0.09	0.41
	DEEP	Diat_IC	0.23	0.18	0.30
		Epiphytes	0.36	0.26	0.47
		Fil_IC	0.08	0.05	0.12
		Fil_INT	0.21	0.11	0.35
		Sed_C	0.12	0.08	0.21
